# A minimum evaluation protocol and stepped-wedge cluster randomized trial of ACCESS Open Minds, a large Canadian youth mental health services transformation project

**DOI:** 10.1186/s12888-019-2232-2

**Published:** 2019-09-05

**Authors:** Srividya N. Iyer, Jai Shah, Patricia Boksa, Shalini Lal, Ridha Joober, Neil Andersson, Rebecca Fuhrer, Amal Abdel-Baki, Ann M. Beaton, Paula Reaume-Zimmer, Daphne Hutt-MacLeod, Mary Anne Levasseur, Ranjith Chandrasena, Cécile Rousseau, Jill Torrie, Meghan Etter, Helen Vallianatos, Adam Abba-Aji, Shirley Bighead, Aileen MacKinnon, Ashok K. Malla

**Affiliations:** 1 Department of Psychiatry, McGill University, Montréal, Québec Canada; 2 ACCESS Open Minds (Pan-Canadian Youth Mental Health Services Research Network), Douglas Mental Health University Institute, Montréal, Québec Canada; 3 Prevention and Early Intervention Program for Psychosis (PEPP), Douglas Mental Health University Institute, Montréal, Québec Canada; 4 Douglas Mental Health University Institute, Montréal, Québec Canada; 5 Department of Epidemiology, Biostatistics and Occupational Health, McGill University, Montréal, Québec Canada; 6 School of Rehabilitation, Faculty of Medicine, Université de Montréal, Montréal, Québec Canada; 7 Centre de recherche du Centre hospitalier de l’Universite de Montreal (CRCHUM), Montréal, Québec Canada; 8 Department of Family Medicine, Community Information and Epidemiological Technologies (CIET) Institute and Participatory Research at McGill (PRAM), McGill University, Montréal, Québec Canada; 9 McGill University Institute for Human Development and Well-being, Montréal, Québec Canada; 10 Department of Psychiatry, Université de Montréal, Montréal, Québec Canada; 11 Centre hospitalier de l’Université de Montréal (CHUM), CRCHUM, Montréal, Québec Canada; 12 School of Psychology, Faculty of Health Sciences and Community Services, Université de Moncton, Moncton, New Brunswick, Canada; 13 Mental Health and Addictions Services, Bluewater Health and Canadian Mental Health Association, Lambton Kent, Ontario, Canada; 14 Eskasoni Mental Health Services, Eskasoni First Nation, Nova Scotia, Canada; 15 ACCESS Open Minds Family and Carers Council, Douglas Mental Health University Institute, Montréal, Québec Canada; 16 Schulich School of Medicine, Western University, London, Ontario Canada; 17 Centre de recherche SHERPA, Institut Universitaire au regard des communautés ethno culturelles, Centre intégré universitaire de santé et de services sociaux (CIUSSS) du Centre-Ouest-de-l’Île-de-Montreal, Montréal, Québec Canada; 18 Public Health Department, Cree Board of Health and Social Services of James Bay, Cree Nation of Mistissini, Québec Canada; 19 Counselling Services, Inuvialuit Regional Corporation, Inuvik, Northwest Territories, Canada; 20 Department of Anthropology, University of Alberta, Edmonton, Alberta Canada; 21 Alberta Health Services, Edmonton Zone, Edmonton, Alberta Canada; 22 Department of Psychiatry, University of Alberta, Edmonton, Alberta Canada; 23 Sturgeon Lake Health Centre, Sturgeon Lake First Nation, Saskatchewan, Canada; 24 Saqijuq Project, Nunavik, Québec Canada

**Keywords:** Youth mental health, Rapid access, Patient-oriented research, Mental health services, Early intervention, Young adults, Adolescents, Indigenous, Canada

## Abstract

**Background:**

Many Canadian adolescents and young adults with mental health problems face delayed detection, long waiting lists, poorly accessible services, care of inconsistent quality and abrupt or absent inter-service transitions. To address these issues, ACCESS Open Minds, a multi-stakeholder network, is implementing and systematically evaluating a transformation of mental health services for youth aged 11 to 25 at 14 sites across Canada. The transformation plan has five key foci: early identification, rapid access, appropriate care, the elimination of age-based transitions between services, and the engagement of youth and families.

**Methods:**

The ACCESS Open Minds Research Protocol has multiple components including a minimum evaluation protocol and a stepped-wedge cluster randomized trial, that are detailed in this paper. Additional components include qualitative methods and cost-effectiveness analyses. The services transformation is being evaluated at *all* sites via a minimum evaluation protocol. Six sites are participating in the stepped-wedge trial whereby the intervention (a service transformation along the key foci) was rolled out in three waves, each commencing six months apart. Two sites, one high-population and one low-population, were randomly assigned to each of the three waves, i.e., randomization was stratified by population size. Our primary hypotheses pertain to increased referral numbers, and reduced wait times to initial assessment and to the commencement of appropriate care. Secondary hypotheses pertain to simplified pathways to care; improved clinical, functional and subjective outcomes; and increased satisfaction among youth and families. Quantitative measures addressing these hypotheses are being used to determine the effectiveness of the intervention.

**Discussion:**

Data from our overall research strategy will help test the effectiveness of the ACCESS Open Minds transformation, refine it further, and inform its scale-up. The process by which our research strategy was developed has implications for the practice of research itself in that it highlights the need to actively engage all stakeholder groups and address unique considerations in designing evaluations of complex healthcare interventions in multiple, diverse contexts. Our approach will generate both concrete evidence and nuanced insights, including about the challenges of conducting research in real-world settings. More such innovative approaches are needed to advance youth mental health services research.

**Trial registration number:**

Clinicaltrials.gov, ISRCTN23349893 (Retrospectively registered: 16/02/2017).

**Electronic supplementary material:**

The online version of this article (10.1186/s12888-019-2232-2) contains supplementary material, which is available to authorized users.

## Background

Mental disorders usually begin before the age of 25 [1] and can persist [2], sometimes in changing forms [3]. They often disrupt the pursuit of the typical social and educational developmental milestones of youth and impose much suffering and societal cost [2, 4, 5]. Mental illness is the leading contributor to disability-adjusted life years among people aged 0–24 years in high-income countries [6]. In Canada, suicide accounts for 20–25% of deaths among 15- to 24-year-olds [7]. Canada’s Indigenous youth suffer exceptionally high rates of mental health problems, substance abuse, and suicide [8, 9].

Adolescence and young adulthood represent a critical window for early detection and intervention because that is when most mental illnesses arise [1] and are presumably most amenable to treatments that can improve long-term outcomes and reduce disease burden [10, 11]. Moreover, longer durations of untreated mental illnesses are generally associated with worse clinical and functional outcomes and greater personal and familial suffering [12–14]. Yet, many youth with mental health problems remain untreated or face delayed detection [15].

In Canada, where 18.5% of youth aged 15 to 24 are affected by mental and substance use disorders [16], a 2012 survey found that only 49.7% of youth with mental health problems and 25.8% of those with substance use disorders had sought professional help in the preceding year [17]. Our systematic review [18] found that help-seeking youth’s pathways to mental healthcare were often complex, stressful/negative (e.g., involving emergency rooms or police) and delay-prone. Across much of Canada, long waiting lists significantly delay evaluation and treatment [19] and youth are also excluded from care based on not meeting specific diagnostic criteria [20]. Canadian youth mental healthcare is also marred by improper transitions between child-adolescent and adult services; a paucity of evidence-informed interventions; fragmented, inefficient and siloed system components [21, 22]; and little involvement of patients and their families in service design and planning, despite growing acknowledgement of the value of such involvement [23–26]. Consequently, services fail to engage and meet the needs of youth and their families.

Addressing these issues is the remit of ACCESS Open Minds, a research network established under the Strategy for Patient-Oriented Research (SPOR) [24] of the Canadian Institutes of Health Research (CIHR). This five-year project draws on the experiences of Australian, Irish and British youth mental healthcare improvement initiatives and previous work on early intervention in psychosis [27–29] Partnering with multiple stakeholder groups (youth and families with lived experience, service providers, researchers, and decision-makers), ACCESS Open Minds developed, and is implementing and evaluating a transformation of services for youth (aged 11 to 25) with all types and severities of mental health difficulties at 14 sites across Canada [30].

ACCESS Open Minds is implementing strategies to increase youth referrals and help-seeking; accelerate response times; provide timely access to appropriate interventions as per established benchmarks; eliminate age-based transitions between services; and engage youth and families. The transformation also involves creating youth-friendly, stigma-reducing, community-based spaces that allow walk-in access to integrated services and supports. It is hypothesized that this transformation will improve the reach, timeliness, appropriateness, quality, acceptability, clinical and functional outcomes, and subjective experience of mental health services. The ultimate goal is to create a sustainable and scalable model for timelier, friendlier, and more effective youth mental healthcare.

### Objectives

This study evaluates the effectiveness of the ACCESS Open Minds model using a multi-method design that includes a minimum evaluation protocol, a pragmatic stepped-wedge randomized trial, a qualitative strategy, and a cost-effectiveness evaluation. This paper focuses on the minimum evaluation protocol and the stepped-wedge trial. The qualitative and economic evaluations will be described in separate publications.

Given the breadth of issues addressed, strategies deployed and populations targeted, we ask the following main questions in this study:

- How and to what extent does ACCESS Open Minds work to identify youth in need and improve their access to high-quality mental healthcare?
- Among which youth groups and in which contexts is the transformation most and least beneficial?

These research questions are operationalized through well-defined primary and secondary objectives and related hypotheses.

### Primary objectives

To determine if the ACCESS Open Minds model:

1. Increases early case identification
  Hypothesis 1: There will be a significant increase in the number of youth seeking help at, or being referred to, study sites over the course of the project.
2. Reduces systemic delay in responding to referrals/help-seeking
  Hypothesis 2: The proportion of youth being offered an initial assessment by a trained clinician within 72 h of referral/help-seeking will increase and be sustained at or above benchmark levels (once attained) over the course of the project.
3. Reduces treatment delay (i.e., the delay between the initial evaluation and the commencement of appropriate treatment)
  Hypothesis 3: Over the course of the project, a higher proportion of youth seeking services at sites will be offered appropriate care/interventions within 30 days (the Canadian Psychiatric Association’s benchmark) [31]. For urgent cases, the guidelines recommend commencing treatment in less than 30 days. This will be examined separately for youth with and without serious mental illnesses.

### Secondary objectives

To determine if the ACCESS Open Minds model:

4. Simplifies pathways to mental healthcare
  Hypothesis 4: Over the course of the project, youth will make fewer help-seeking contacts in the previous 12 months before accessing services at project sites.
5. Improves outcomes for youth served at project sites
  Hypothesis 5: The clinical, social-vocational and subjective functioning of youth will significantly improve over the course of their follow-up. An additional objective is to determine which outcome domains are most impacted by treatment. The lengths of follow-up will vary depending on youth’s needs, severity of illness, etc.
6. Satisfies youth and their families
  Hypothesis 6: At least 75% of service users and their families/carers will be satisfied with services and service providers at all completion time-points.

## Methods

### Setting/sites

ACCESS Open Minds’ 14 sites represent pan-Canadian variations in geography, culture, resources, and population density [30] (see Table 1). Together, they serve youth in urban, rural, and remote communities; non-Indigenous and Indigenous youth; Anglophone and Francophone youth; and groups with particular vulnerabilities (immigrants, refugees, ethnic minorities, state-protected youth, homeless youth, and first-year university students). Ten sites cover specified catchment areas, strengthening the representativeness of the youth populations they serve.

**Table 1:** ACCESS Open Minds Site Descriptions (A list of study sites with addresses can be obtained from the trial registry)

Site (prominent languages of the milieu)	Province	Youth Population^1^	Target N of youth expected to consent to research^2^	Notable features of youth population, if any	Service system features
URBAN SITES					
PEER Saint John (English)	New Brunswick	11,085	277	Many low socio-economic status, many NEET^3^	Mental health day-treatment centre
Dorval-Lachine- Lasalle (French, English)	Québec	18,530	864	28% speak languages other than English/French	Primary care centre offering health and social services to specified geographic catchment
RIPAJ-Montréal Homeless Youth Network* (French, English)	Québec	1000*	93	Homeless, many NEET	Network of community organisations; and primary and tertiary public health and social service settings
Parc-Extension (English, French)	Québec	5065	236	Large numbers (over 60%) are visible minority and immigrant	Primary care centre offering health and social services to specified geographic catchment
Edmonton (English)	Alberta	17,010	700	Many homeless, Many NEET	Governed by single authority that provides health care to entire province
University of Alberta* (English)	Alberta	8000	329	First year university students	Student services at the university
SEMI-URBAN / RURAL					
Caraquet, Acadian Peninsula (French)	New Brunswick	555	39		Mobile team and community centre
Chatham-Kent (English)	Ontario	17,355	865	Also serves two neighboring First-Nation communities	Community-based youth services hub; Key partners are public health and addictions program and a community mental health organization.
INDIGENOUS / REMOTE					
Eskasoni First Nation (Mi’kmaq, English)	Nova Scotia	1025	–		Mental health centre (division of health centre) accountable to the Band Council
Elsipogtog First Nation (Mi’kmaq, English)	New Brunswick	839	–		Health centre and youth space accountable to the Band Council
Cree Nation of Mistissini (Cree, English)	Québec	1015	–		Network of services funded by Cree Health Board and local Band Council
Puvirnituq (Inuktitut, English)	Québec	535	–	Remote, Northern	Lay health workers in collaboration with Saqijuq, a community youth diversion initiative
Sturgeon Lake First Nation (Plains Cree, English)	Saskatchewan	350	–		Youth Space with mobile ACCESS clinician services
Ulukhaktok (Inuvialuit, English)	Northwest Territories	105	–	Remote, Northern	Lay health workers in collaboration with Inuvialuit Regional Corporation^4^

^1^Based on estimate for number of youth aged 10 to 24 from the Census Profile, 2016 Census, Statistics Canada ^2^ This column represents the expected number of youth who will consent to research at each site. This number represents 60% of the total number of youth who are projected to receive services at each site. This projected number was arrived at using each site’s known youth population and estimates of youth mental health help-seeking prevalence and unmet needs from the Canadian Community Health Survey – Mental Health (CCHS-MH; 2012). Although Indigenous communities had not been included in the CCHS-MH, the same formula was used to arrive at minimum target numbers for the Indigenous community sites, knowing that these would be under-estimates given the expected higher prevalence of mental health help-seeking in Indigenous contexts. These minimum estimates for Eskasoni First Nation, Elsipogtog First Nation, Cree Nation of Mistissini, Puvirnituq, Sturgeon Lake First Nation and Ulukhaktok were 68, 56, 44, 23, 17 and 5, respectively. The CCHS-MH based formula was also not used for RIPAJ and University of Alberta (marked with *) which are not catchment-area based sites. ^3^ NEET = Not in employment, education or training ^4^ Inuvialuit Regional Corporation (IRC) is an Indigenous Organization currently under-going self-government negotiations. IRC does not directly deliver mental health services in the Inuvialuit Settlement Region but delivers many social, wellness and cultural programs that supplement services provided by the Government of Northwest Territories

Six sites are in Indigenous communities, where populations tend to be young [32]. In some Indigenous communities, youth are reported to have very high rates of suicidality, addiction, violence, school drop-out, unemployment, and involvement with justice and youth protection systems [33]. It has been proposed that these problems stem from and are compounded by the cultural fragmentation and inter-generational trauma inflicted by brutal colonial and government policies [34]. The situation is exacerbated by varying combinations of geographic isolation (e.g., two sites are only accessible by air and boat); poverty; the inadequacy of health and psychosocial services and associated funding; and service providers’ unfamiliarity with local histories, languages and contexts [35].

### Study intervention

At each site, the ACCESS Open Minds “intervention” transforms services [21, 22] to provide:

1. *Early case identification* through targeted outreach, community awareness, etc., so that more youth self-refer or are referred sooner (see online early identification guide) [36].
2. *Rapid, engaging access*, offering an initial evaluation within 72 h in a non-emergency, community-based environment. To this end, a trained “ACCESS Clinician” is deployed to conduct initial evaluations; involve family members in assessment; and link youth with services tailored to their needs and preferences. Rapidity is also ensured through the plurality of portals of access, including direct walk-in; the elimination of referral or administrative requirements; and the use, where appropriate, of technologies like social media, helplines, etc.
3. *Appropriate care* in the form of evidence-informed, illness-appropriate interventions offered within 30 days of initial evaluation (per Canadian Psychiatric Association benchmarks) [31]. Because youth can present with non-specific, overlapping symptoms, treatment planning is guided by youths’ self-reported distress and social-vocational functioning and clinicians’ impressions of problem severity, rather than specific diagnoses. Provided in youth-friendly, non-stigmatizing, and recovery-oriented environments, care is focused on youth’s own goals. Where appropriate interventions are unavailable, site staff connect youth to external services/specialists.
4. *Continuity of care*, to ensure that youth receive appropriate care for as long as needed. This is achieved by fostering collaboration between services, stakeholders, sectors, and disciplines to eliminate jarring age-based transitions and to reduce or smoothen transitions between other services youth need, e.g., from primary to specialized care.
5. *Youth and family/carers engagement*, to ensure their active involvement, in keeping with the SPOR vision of valuing lived experience. This entails including youth and families in network- and site-level service design, oversight, and hiring committees; seeking their inputs in designing youth spaces; offering individualized menus of interventions, flexible appointment times and, when possible, choices of treatment venues; and training clinicians in youth-friendly, strengths-affirming approaches.

At all sites, transformation began with a community mapping [37–39] exercise that identified, linked up and, where possible, co-located all youth-focused resources in the community (see online guide) [40]. Additional capacity was integrated through hiring an ACCESS Clinician and other requisite staff, training, and external partnerships (e.g., with the national helpline, Kids Help Phone). Additional details about the model and its operationalization appear in our prior open-access publication [30].

Although a dynamic process, service transformation is deemed to commence at each site with: (a) the finalization of a site-specific service transformation plan (on the basis of which a contract is signed and funds are allocated) with clear activities outlined to achieve each service objective; (b) the deployment of the ACCESS Clinician; and (c) the training of key site staff in ACCESS Open Minds principles and evaluation protocols.

Core strategies common to all sites’ transformation plans include deploying an ACCESS Clinician; responding to help-seeking/referrals within 72 h; and creating a youth-friendly physical space. Relevant evidence and local conditions inform additional site-specific strategies. For instance, twice-yearly early identification activities may target all youth at sparsely populated Indigenous sites, but only potential referral sources (e.g., schools) at more populous urban sites. The relevance, acceptance and ownership of each site’s service transformation plan are assured through broad stakeholder engagement.

### Study design

The impact of ACCESS Open Minds is being evaluated using a multi-method design that includes a minimum evaluation protocol at all sites, and a stepped-wedge cluster randomized trial at six sites.

*Minimum evaluation protocol*: Our minimum evaluation protocol — developed collaboratively in consultation with youth, families, researchers, and clinicians — is designed to collect data that address our primary and secondary hypotheses (see Table 2). These include measures/indicators of: service contexts (e.g., numbers of youth in the catchment area); service users (e.g., number of males accessing services); service provision/processes (e.g., portals of entry available, types of early identification strategies deployed, etc.); and outcomes/impacts (e.g., time between help-seeking and initial evaluation, satisfaction with services, reduction in distress, etc.).

**Table 2:** Summary of assessment tools selected for the study

Instrument	Informant(s)	Key construct	Subscales	Measurement time(s)^a^
*Description of participant sample*				
Customized ACCESS OM sociodemographic questionnaire	Youth; Clinician; Administrative records	Sociodemographic characteristics	None	Intake, Months 3, 6, 9, 12 and 34/end of services
Custom checklist	Youth, Clinician	Presenting concerns	None	Intake
GAIN-SS^x^	Clinician-administered youth report	Clinical symptoms	Internalising problems, externalizing problems, substance use disorders, crime/violence	Intake, Months 3, 12 and 24/end of services
Columbia-Suicide Severity Rating Scale (C-SSRS): Screener^x^	Clinician	Suicidal ideation and behavior over past month plus one item re past suicidal behavior	None	Intake, Months 1, 3, 6, 9, 12 and 24/end of services
*Service-level improvements*				
Customized services forms	Clinician; Administrative records	Number of referrals (Hyp. 1); Number offered evaluation 72 h after referral request (Hyp 2); Number offered appropriate treatment within 30 days after completion of initial evaluation (Hyp. 3)	None	Intake, Month 1; Services forms repeated at months 1, 3, 6, 9, 12, and 24 or at end of services to document services needed and offered
CCHS-MH 2012 questions related to services sought and received	Clinician-interview with youth and/or family/carer, plus any available records	Number of help-seeking contacts made for problems with mental health/substance use and whether or not services were received in previous 12 months (Hyp. 4)	None; can examine type of contacts (formal, informal, etc.)	Intake
*Clinical improvements*				
Kessler-10 Psychological Distress Scale (K-10)	Youth	Unidimensional factor of distress as being linked to mental ill-health and varied diagnoses (Hyp. 5)	Cut-offs based on overall score (e.g., mild distress)	Intake, Months 1, 3, 6, 9, and 12
Clinical Global Impression of Severity (CGI)^x^	Clinician	Clinician’s rating of severity of youth’s presenting problem over time (Hyp. 5)	None	Intake, Months 1, 3, 6, 9, 12 and 24/end of services
Self-rated health and self-rated mental health (SRH-MH)	Youth	Self-assessment of health and mental health, each on a single 5-item Likert scale (Hyp. 5)	None	Intake, Months 1, 3, 6, 9, and 12
*Functional improvements*				
Social and Occupational Functioning Assessment Scale (SOFAS)^x^	Clinician	Social and vocational functioning on a 0 to 100 scale (Hyp. 5)	None	Intake, Months 1, 3, 6, 9, 12 and 24/end of services
*Subjective/Well-being improvements*				
Goals tool inspired by the Goals-based Outcome (GBO) tool*^x^	Youth	Self-rated progress on 3 chosen goals (Hyp. 5)	None	Intake, Months 3, 6, 12 and 24/end of services
Outcome Rating Scale (ORS)^x^	Youth	Overall outcome/ functioning/ well-being (Hyp. 5)	None; Items related to overall; individual; interpersonal; and social outcomes can be examined separately	Intake, Months 6, 12 and 24/end of services
World Health Organisation Quality of Life – Brief (WHOQOL)*	Youth	Self-assessment of quality of life (Hyp.5)	Four domains: physical health, psychological health, social relationships, and environment	Intake, Months 6, 12 and 24/end of services
*Service experiences*				
OPOC^x^	Youth, family	Satisfaction (Hyp. 6)	Access to service, services provided, participation/ rights, therapists/support workers/staff, environment, discharge, recovery outcome, service quality	Months 1, 3, 6, 9, 12 and 24/end of services
Session Rating Scale (SRS)^x^	Youth	Working Alliance (Hyp. 6)	None; Items related to relationship; goals or topic; approach or method; and overall can be examined separately	Intake, Months 6, 12 and 24/end of services
Youth Efficacy/Empowerment Scale (YES)*	Youth	Empowerment	Self, services, system	Intake, Months 3, 6, 9, 12 and 24/end of services

Hyp. = Hypothesis; OPOC = Ontario Perception of Care tool; GAIN-SS = Global Appraisal of Individual Needs –Short Screener

*Completed at select sites and not all ACCESS Open Minds sites

^a^As youth presenting with different concerns may need varying lengths of follow-up, the assessment protocol is accordingly applied. For example, if the young person receives a follow-up at an ACCESS Open Minds site for 6 months, the SOFAS will be administered at Intake, Month 1, Month 3 and Month 6. However, if they receive follow-up for just 1 month, the SOFAS will only be administered at Intake and Month 1

^X^Irrespective of length of follow-up, the following measures are to be administered at the end of follow-up: Demographic Information; SRS; YES; OPOC; WHOQOL; Services Form; SOFAS; CGI; C-SSRS; GAIN-SS; GBO-inspired tool

Measures/indicators were selected based on the following priorities:

1. Sustainability, to increase the likelihood of the protocol’s continued use after the research project, consistent with the ideal of measurement-based care [41–43].
2. Wide applicability, so that measures/indicators address research objectives; inform treatment and quality improvement; and enjoy buy-in, especially from sites that had little prior research or evaluation experience. To ensure wide applicability, we chose measures/indicators targeting multiple informants, including youth, families/carers, and clinicians/service providers.
3. Appropriateness, to choose measures shown to have adequate psychometric properties in varied Canadian youth populations. Some measures were chosen because they had been used in population-wide surveys (Canadian Community Health Survey-Mental Health [44] and Aboriginal People’s Survey [45]. This will allow the comparison of data from our sites with corresponding population-level data. When several options were available, shorter, more user-friendly measures were chosen to enhance feasibility and acceptability.

Data are collected throughout the study period, and over-time comparisons will be conducted at the network and individual site levels. At some sites, we will also conduct pre-post comparisons.

**Fig. 1:**
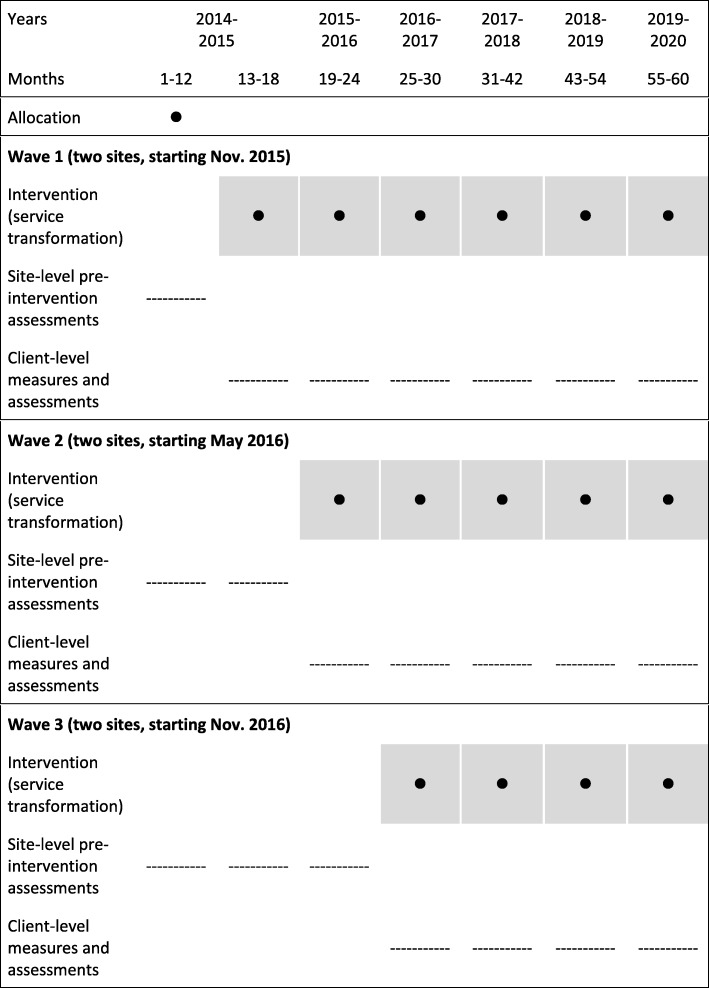
Flowchart of trial using SPIRIT flowchart recommendations

**Fig. 2:**
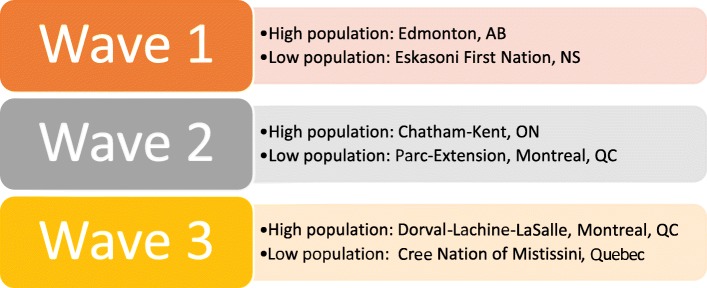
Configuration of waves after randomization in April 2015

*Stepped-wedge trial*: Six sites were selected to participate in a randomized stepped-wedge trial [46, 47] wherein the intervention (a transformed service) was sequentially rolled out in three clusters/waves, starting six months apart. Sites were rank-ordered based on population, and the first three ranked sites were grouped as high and the last three ranked sites were grouped as low population. Thus stratifying by population size, two sites, one high-population and one low-population, were randomly assigned to each wave (see Fig. 1). At an event attended by representatives from all six sites, the randomization assignment was conducted by drawing chits from separate urns containing names of large and small sites (see Fig. 2).

All stakeholders deemed the stepped-wedge design preferable on ethical grounds because it deployed the transformation at all sites, rather than having some sites serve solely as “controls.” For their capacity to generate high-impact evidence (like traditional RCTs), stepped-wedge trials are increasingly used to evaluate complex service-based and public health interventions [48]. They involve the random and sequential crossover of waves from the control condition to the intervention until all waves are exposed.

The lack of concealment of the allocation of the intervention can introduce selection biases to stepped-wedge trials [49–51]. It may also be difficult to separate the effects of the intervention from changes that simply happen over time. However, this can be countered statistically [52]. Such trials are prone to logistical constraints, particularly with complex, multi-component interventions [49–51]. For instance, the intervention may commence or attain adequacy at a site later than when its cluster was randomized to begin the intervention due to external events.

Per the stepped-wedge design, data collection continues throughout the study at all sites, so that each cluster contributes observations under both control and intervention conditions. All six sites thus act as controls whilst also undergoing transformation. Refer to Additional file 1: SPIRIT Checklist [53, 54], which lists where specific trial details can be found in this paper.

### Project management and participation plan

Our earlier publication [30] outlines the project’s governance structure and processes. Research is guided by a Research Advisory Group comprising representatives from all sites; network-level youth and family/carers council representatives; and network-level researchers. Overall guidance is provided by a Steering Committee of four external researchers who, together, have expertise in youth mental health, primary healthcare, Indigenous issues, and biostatistics.

At each site, evaluations are conducted by local staff, who were trained by the Montréal-based central office team, tasked with supporting sites and operationalizing the project. Site staff also receive booster training and ongoing support. At regular sessions where all sites participate, multiple raters rate the same scenarios independently on two clinician-administered measures (see Table 2), discuss ratings, and establish consensus guided by experts. These sessions help raters calibrate themselves in relation to a scale’s anchors and ensure that scales are reliably rated across sites.

Videos [55–57] were created to explain the project, the role of evaluations, and informed consent to site staff and youth. Videos featuring actors were also developed to demonstrate best practices for introducing evaluations and informed consent to youth. The project’s national youth council created and shared engaging, youth-friendly posters describing the project and its research/evaluation strategy.

### Participant recruitment, consent and withdrawal

The sites will recruit youth for a period of 3–3.5 years until September 2020. The first participant was enrolled in the study in July 2016. Participants include youth and, where possible, their families/carers.

While details vary across sites, the general recruitment procedure is for the ACCESS Clinician or another staff member to identify new referrals to a research assistant/staff who explains the project to them and seeks written informed consent (see Additional file 2 for sample consent form). Consenting youth are enrolled into the study.

The protocol recommends approaching youth for written consent at the time of intake. However, recruitment can be deferred if youth are in crisis situations or too busy. For minors below the age of 14 or youth with diminished capacity, consent is sought from a parent/legal representative along with assent from the young person. Provincial/institutional regulations vary in terms of policy around the need for consent from a parent/legal representative in the case of youth between the ages of 14 and 18 (see Declaration about “Ethics approval and consent to participate”).

Consent is also sought from family members for their participation in family-focused assessments. Signed forms are filed securely in locked cabinets and copies are given to participants.

Consent is voluntary and can be withdrawn at any time, with no impact on access to services or the quality of care. Consent simply allows data from measures completed by youth and from their health administrative records to be recorded and analyzed for the research project.

Following youth feedback, a consent form (see Additional file 2) was created that, while modeled on standard ethics guidelines, minimized length and legalese. It describes the services offered, details the research/evaluation component, and clarifies that consenting to research is not a prerequisite to receiving services. It thus respects the project’s philosophy of integrating care and evaluations. Across sites, the wording or structure of consent forms varies to respect local ethics procedures and cultural considerations. Sites have also developed recruitment strategies suited to their context and youth population. Some sites offer honoraria/compensation to youth for time and travel costs incurred in completing assessments, and others often meet youth outside, in the community or a café, to complete assessments, etc.

*Inclusion and exclusion criteria*: To recruit as representative a sample as possible of *all* youth served, inclusion criteria are broad and exclusion criteria minimized. For enrollment into research/evaluation, youth must be aged 11 to 25 years and be seeking help for a mental health and/or substance-related problem. Youth who received services at a site before its transformation are eligible if they have not received any services for six months or longer. This was done to ensure that the study of outcomes is restricted to those receiving transformed services. Individuals younger than 11 years or older than 25 years; with a diagnosed intellectual disability; with a history of organic brain damage; and/or deemed unable to consent are not eligible. For family members/carers to be included in the research, they must be connected to a youth receiving services at a site and provide informed consent.

### Outcome measures

Outcome measures are summarized in Table 2 and their psychometric properties in Additional file 3. Measures that were available only in English were translated into French with forward and back translation and a focus on conceptual equivalence [58, 59]. All purpose-designed forms are available in English and French. Measures of clinical, functional and subjective outcomes are administered at baseline and repeated for those who receive further treatment.

*Sample description*: Socio-demographic variables like sex, age, housing situation, ethnicity, and immigration background are obtained from a self-report form, clinical interviews, or records. Such data are crucial given the known social determinants of mental healthcare access and outcomes.

Where possible, items were picked to allow population-level comparisons, e.g., a Canadian census indicator of visible minority status [60] to help determine whether sites serve as many minority youth as the ethnic composition of their catchment populations would suggest they should.

A clinical profile of service recipients is created using a purpose-designed checklist of presenting concerns, the Global Assessment of Individual Needs-Short Screener [61], and the Columbia-Suicide Severity Rating Scale screener [62], among other tools..

*Service-level outcomes*: Forms were purpose-designed to record information about sites’ performance on key indicators pertaining to our three main hypotheses (increase in number of youth assessed, evaluation within 72 h, and adherence to the 30-day benchmark).

Data from these forms will also help identify barriers to meeting project benchmarks (e.g., delay due to lack of an on-site psychiatrist) and site-level quality improvement (e.g., information on referral sources will help tailor early case identification activities). Secondary questions can also be asked, e.g., about whether self-referrals increase over the course of the project. Staff will record on these forms up to three services needed by youth following the initial evaluation and services received by them 30 days later, which will serve as our measure of “appropriate” care.

Items taken from the Canadian Community Health Survey-Mental Health (CCHS-MH) 2012 survey [44] are used to determine whether youth are first-time help-seekers and the number and types of help-seeking contacts they make before entering a site (Hypothesis 4).

*Clinical outcomes*: To gauge the extent of clinical improvements, both self-rated and observer-rated measures are used. This includes the Kessler Psychological Distress Scale (K10) [63], a self-report measure of distress, and two self-rated single-item measures of physical and mental health [64, 65]. These measures are quick, easy to complete, validated, and used widely in clinical and population surveys, including in Canada [66]. The K10 is also used in Australian youth mental health hubs [67, 68].

Clinicians or trained research staff rate the severity of youth mental health presentations using the single-item Clinical Global Impression (CGI) scale [69]. Because youth present with highly diverse problems, anchor descriptions from the trans-diagnostic version [70] of the CGI were adopted. At all time points after the initial evaluation, staff also rate improvement in symptoms using the single-item CGI-Improvement. The CGI was chosen over more elaborate measures to increase clinician buy-in and rates of completion.

*Functional outcomes*: To examine if transformed services improve functioning, the Social and Occupational Functioning Scale (SOFAS) completed by clinicians or research assistants will be used. The SOFAS [71] is rated on a 1-to-100 continuum, without considering the severity of symptoms. In addition, the work/school status of youth is also being recorded.

*Subjective outcomes and well-being*: Three measures assess youth’s subjective progress and well-being following intervention — outcomes that were prioritized by youth members of the network.

The minimally burdensome Outcome Rating Scale assesses four dimensions of life functioning that indicate the success of therapeutic interventions [72]: individual well-being (measuring personal or symptom distress); interpersonal well-being (measuring functioning in intimate relationships); social role (measuring satisfaction with work/school and non-familial relationships); and overall well-being. At select sites, youth also complete the World Health Organization Quality of Life (WHOQOL-BREF) [73], a quick measure of perceived quality of life.

Given the larger goal of evaluating impacts for all youth across settings, most measures in our protocol are nomothetic. However, being a patient-oriented research project, it was thought important to include an idiographic measure inspired by the Goals Based Outcome (GBO) [74]. It allows youth to rate their progress toward three self-identified, personally salient goals during their follow-up. Family members/carers are also invited to choose and rate progress towards three goals in relation to their loved ones’ services. Pooled data on goal attainment will help evaluate the effectiveness of services, as has been done in the United Kingdom with the GBO [75–77].

*Service experiences*: To assess the extent to which ACCESS Open Minds transforms cultures of care, the youth and family versions of the Ontario Perceptions of Care (OPOC) [78] (a validated tool developed in Canada) will be used to assess satisfaction with the accessibility, quality and other aspects of services. Therapeutic alliance will be assessed using the Session Rating Scale (SRS) and youth’s perceptions of empowerment and engagement using the Youth Efficacy/Empowerment Scale [79]. These tools complementarily assess alliance with individual care providers (SRS) [80] and overall satisfaction with a service (OPOC).

### Data collection procedures

At all sites, data collection by trained clinical and research staff begins with referral and continues throughout follow-up, the length of which varies according to the needs of youth. Research assistants need 1 to 1.5 h to complete initial assessments and about 30 min for follow-up assessments. They have access to staff for help with any emergent crises.

In this study, “data” refers to data obtained directly from consenting individuals (e.g., self-reports) and non-identifiable data from pertinent health administrative records obtained with institutional approval. A secure electronic data capture and data management system makes the research/evaluation protocol accessible online from anywhere. Project staff, youth, and families can use the system to complete measures and forms on any device (desktop, tablet, or phone). Self-administered measures and forms can be emailed to youth/families for future completion on a user-friendly interface. The entire system — measures, forms, and interface — is bilingual (French and English). An integrated scheduling function facilitates follow-ups at time points as per the protocol. In case of connectivity issues, data can be collected offline and uploaded later. People who prefer completing measures/forms on paper can do so. Whether collected on paper or digitally, all data are entered into the electronic system.

Once data are entered, customized reports can be easily extracted to inform care in real time; e.g., clinical progress reports can support intervention decisions and goal attainment reports can help demonstrate improvement to youth. Dashboards within the software can be customized to display frequently sought information.

We expect to generate valuable insights into customizing and deploying electronic data capture and management systems in diverse real-world service and research settings. We will also seek to understand whether such a user-friendly system promotes measurement-based mental healthcare, which, despite its well-acknowledged value [41–43], is rare or inconsistent in Canada [81].

Periodic audits will be conducted at all sites to verify adherence to ethical and recruitment procedures, and to verify accuracy of data being collected. This audit will be undertaken by research team members based in a central coordinating office in Montréal. Central office staff also will regularly review electronic data to flag double data, data values not being in range, etc.

### Ethical considerations

Having obtained ethics approval from pertinent institutional bodies (see Additional file 4), the study is following appropriate regulations and standards regarding data privacy, confidentiality, storage, and security (see Declaration about “Ethics approval and consent to participate”). A checklist is being used to record adverse events (e.g., death by suicide, accidents, etc.), which are communicated to site and central institution ethics committees and to the project steering committee.

This study is being conducted according to Good Clinical Practice [82]. Appropriate procedures include informed consent from individuals, and institutional approval for access to pertinent non-identifiable health administrative records. Data are stored securely, and confidentiality of data will be ensured. A steering committee has been set up to oversee the project and provide inputs to the team of investigators.

ACCESS Open Minds acknowledges Canada’s problematic history of academic and administrative research on Indigenous peoples (outlined in the 1996 Report of the Royal Commission on Aboriginal Peoples) [83]. We follow the Ownership, Control, Access, and Possession (OCAP™) principles [84] and other precepts of the guidelines for Research Involving the First Nations, Inuit and Métis Peoples of Canada from the Tri-Council Policy Statement on Ethical Conduct for Research Involving Humans [85].

Within the ACCESS Open Minds project, OCAP principles are being implemented at all levels. Specifically, community leadership and the ACCESS Open Minds Indigenous Council have played integral roles in partnering with Indigenous communities, guiding conversations about the implementation of the research and evaluation protocol, and bringing together important voices and perspectives from the community. The OCAP principles are underscored in all research and partnership agreements held between the ACCESS Open Minds project leads and Indigenous communities. Most importantly, key decisions– from youth space design to how data will be analyzed and interpreted -- are being guided by Indigenous site teams, including the site team leader, Elders, youth, and other interested community members. OCAP also addresses the issues of privacy, intellectual property, data custody, and secondary use of data.

In ACCESS Open Minds, following Tri-council guidelines for research involving Indigenous peoples [85] is being seen not merely as a requirement, but as congruent with Indigenous self-determination and full-spectrum stakeholder engagement, both of which are critical in effecting lasting change in youth mental health outcomes in Indigenous communities. Finally, Indigenous communities participating in ACCESS Open Minds also respect the Jordan’s Principle [86] that guarantees equitable access to healthcare services for Indigenous children, regardless of the healthcare jurisdiction in which they live or access services.

### Sample size estimation

To arrive at the targeted sample size for catchment-area based sites (see Table 1), we used CCHS-MH-estimated percentages of the province's (a) youth who had a mental health/substance use disorder; (b) youth with mental disorders who sought help, and (c) youth with unmet needs for mental healthcare [87]. These were prorated to the site catchment’s youth population (i.e., number of youth aged 10 to 24 based on the 2016 Census [88]).

The estimate for unmet needs was added in furtherance of our primary objective of increasing case identification and thereby reducing untreated prevalence. Sites were to target reducing 20% of the unmet needs estimate per year in the project. Estimates for non-catchment area-based sites and for Indigenous community sites were arrived at in consultation with the sites.

The study protocol recommends that sites actively conduct outreach and other early identification activities; not turn away any youth; and approach all youth entering services for consent for research. At sites that have never participated in research, the percentage of youth who will provide consent cannot be estimated. It is therefore difficult to predict exactly how many youth will ultimately be included in our study. However, preliminary numbers from ongoing data collection suggest that 60% of youth approached will provide consent. It is estimated that the project will serve at least a total of 6029 youth. With a 60% consent rate, the estimated number of research participants from all 14 sites by the end of the project will be 3616. This should provide sufficient number or sample to answer the primary hypotheses.

### Statistical plan

Every attempt will be made to collect outcome data from all participants, including those who do not complete treatment. Youth will be considered as having a serious mental illness if they attain the relevant scale-specific cut-off scores on the K-10, SOFAS and/or the CGI-severity scale.

*Pre-post minimum evaluation protocol*: For each site, general linear models with repeated measures will be used to analyze differences over time per site (for hypotheses pertaining to increased referrals, evaluations within 72 h, treatment within 30 days, number of prior help-seeking contacts, and satisfaction with services) and per youth (at baseline and at follow-up timepoints for the hypothesis pertaining to improvements in functioning). Generalized estimation equations will be employed for parameter estimation. Respective baseline values will be added to the model as a covariate. Additional analyses will include examining hypothesis 3 separately for youth with and without serious mental illnesses, examining inter-site heterogeneity, and identifying baseline predictors of response to treatment. Finally, outcomes will be examined separately for large urban, small urban/rural and Indigenous contexts.

*Trial*: Youth demographics and clinical data will be described using means and standard deviations and trial waves will be compared using t-tests or non-parametric tests for quantitative characteristics. The effect of the interventions on the three primary and all secondary outcomes will be quantified using mixed or multi-level models to integrate cluster/wave and time effect. The unidirectional crossover design of the stepped-wedge trial allows us to test the effect of the intervention at both within- and between-cluster levels. Mixed models allow stratification (e.g., by population sizes); inclusion of missing data with the principle of intention to treat; and adjustment for baseline differences.

### Dissemination plan

Findings will be disseminated to various stakeholder groups via appropriate channels, including our project’s website (www.accessopenminds.ca); user-friendly reports, charts, and slide shows for youth, families, sites, and decision-makers; and conference presentations and peer-reviewed journal publications for scientific audiences, including a publication outlining findings pertaining to our primary hypotheses. Dissemination of findings from Indigenous communities will respect OCAP™ principles [84]. Authorship guidelines for the project are available upon request and fall under the purview of the project’s national publications committee.

## Discussion

ACCESS Open Minds delivers and evaluates a large-scale innovation in youth mental health services that adds to, leverages and transforms extant services and resources. While critical additional human, training and material resources are required, the project does not create entirely parallel services distinct from Canada’s publicly funded healthcare system [89]. The project’s broad scope includes youth with widely varying mental healthcare needs from highly diverse geographic, cultural, economic and socio-demographic backgrounds [90–96]. An additional strength is that the ACCESS Open Minds model is informed by multiple relevant stakeholders, including youth, families/carers, researchers, clinicians, Indigenous communities, community organizations, and policy−/decision-makers.

Should its innovative, stakeholder-informed methods and strategies yield clear positive impacts within five years, ACCESS Open Minds can serve as a scalable, sustainable model for transformational change in the organization and delivery of youth mental healthcare in Canada and beyond.

Our multi-method research will show the extent to which the transformation increases and accelerates the early identification, assessment, and appropriate treatment of youth with mental health care needs; and improves pathways into care, satisfaction with services, and a range of mental health outcomes.

Our quantitative analysis will examine impacts over time at the network-wide and individual site levels. This, along with our planned qualitative analyses, will help us understand whether and why the transformation fades, intensifies, or differs across communities. Such insights will inform the applicability of the ACCESS Open Minds model across jurisdictions and will have significant policy implications.

Our carefully chosen metrics strengthen the internal validity of our future findings. Their external validity is enhanced by the diversity of our sites (many serving treated incidence samples) and populations; the investigation of multiple service variables; and the situating of our transformation in “real system, real resources” contexts.

Finally, the development and implementation of our research strategy offer valuable insights into: academic-stakeholder research partnerships; the integration of evaluations in settings that have limited experience with research/evaluations; and the building of research capacity among diverse stakeholders.

### Limitations

The potential of our stepped-wedge trial is limited because only six, very diverse sites were randomized to only three clusters. More, larger, and more similar clusters would have yielded a more robust trial [49–51]. Moreover, the dynamic, real-world nature of the transformation has posed practical challenges that impeded strict adherence to the timelines of our stepped-wedge protocol.

Two Indigenous sites, Ulukhaktok and Puvirnituq, were unable to follow the Minimum Evaluation protocol described in this paper because of cultural considerations, local constraints and preferences. They have also adopted a model that relies on local community workers to promote mental health literacy and wellness, and connect youth in need to supports, in lieu of hiring an “ACCESS Clinician”.

Across sites, the hesitancy of some inexperienced staff members to approach youth for consent could suppress sample sizes. The sheer diversity of local contexts and staff may result in reduced inter- and intra-site uniformity in recruitment and research assessment practices. This is being mitigated by creating a community of practice, conducting inter-rater reliability sessions, and providing training and support.

Our focus on evaluating the service transformation model in its entirety limits our ability to draw conclusions about the effectiveness of specific components. Our sample size and its distribution across 14 sites may preclude sophisticated analyses of the transformation’s differential impacts on different mental health problems. This will be mitigated by asking select questions about whether the model has different effects on presenting problems of differing gravity (mild-moderate versus serious).

Because the ACCESS Open Minds model involves the context-sensitive application of strategies to attain a set of five common objectives, the processes involved in service transformation and the extent to which objectives are attained can vary across sites. This is likely to influence the overall results. The actual processes of transformation at each site are being recorded and will be used to interpret results and to conduct additional explanatory analyses. Thus recording transformation processes will also provide rich, useful implementation data from diverse contexts that can inform similar initiatives in the future.

Finally, because data will be collected for different durations at different sites and for relatively modest lengths of time post-transformation, it will not be possible to assess the long-term site- or network-level impacts of service transformation.

## Conclusion

Data from our research strategy will help assess, refine and scale up the ACCESS Open Minds model and generate concrete evidence to advance youth mental health services research. The collaborative development of our research strategy highlights the need to actively engage all stakeholders and address unique considerations in designing evaluations of complex healthcare interventions. Our participatory, integrated knowledge translation approach involved all key stakeholder groups in identifying and shaping research priorities, processes, methods, and measures. Such involvement, unprecedented in a project of this scale, can inspire other patient-oriented research undertakings. Our use of an electronic data capture and management system is significant given the marked underuse of such systems in Canadian mental health services and health services research [81, 97]. By deploying a common data management system and evaluation protocol in diverse settings, this project will also promote much-needed consensus on using common measures, indicators, and data collection strategies across youth mental health services in Canada. Finally, in executing and evaluating real, transformative actions, ACCESS Open Minds has the potential to catalyze practice- and policy-level change.

## Additional files

Additional file 1: SPIRIT 2013 checklist for ACCESS Open Minds. (DOCX 53 kb)

Additional file 2: Model of consent form used in the ACCESS Open Minds project. (DOCX 54 kb)

Additional file 3: Psychometric properties of study instruments. (DOCX 20 kb)

Additional file 4: Listing of ethics boards; age of research consent and need for parental consent for each site in the ACCESS Open Minds project. (DOCX 15 kb)

## Abbreviations

CCHS-MH: Canadian Community Health Survey-Mental Health
CGI: Clinical Global Impression
GBO: Goals Based Outcome
K10: Kessler Psychological Distress Scale- ten-item version
OCAP: Ownership, Control, Access, and Possession principles
RCT: Randomized controlled trial
SOFAS: Social and Occupational Functioning Scale
SPIRIT: Standard Protocol Items: Recommendations for Interventional Trials
SPOR: Strategy for Patient-Oriented Research
