# Supplementary material for: A minimum evaluation protocol and stepped-wedge cluster randomized trial of ACCESS Open Minds, a large Canadian youth mental health services transformation project
Source: BMC Psychiatry. 2019 Sep 5;19:273. doi: 10.1186/s12888-019-2232-2 (PMC6729084; doi:10.1186/s12888-019-2232-2)
Supplement: Supplementary file 2 — Model of consent form used in the ACCESS Open Minds project. (DOCX 54 kb) [file 12888_2019_2232_MOESM2_ESM.docx]

We welcome you to ACCESS Open Minds (INSERT SITE NAME), a service for young people aged 11 to 25 years with mental health concerns. At ACCESS Open Minds (INSERT SITE NAME), we want to make sure that young people receive appropriate evaluation, and mental health services, supports and care, as quickly as possible.

Additional file 2: Model of consent form used in the ACCESS Open Minds project

Sites have implemented adapted versions of this based on their local ethics requirements. Please contact the corresponding author if you are wish to adapt this consent form.

**The care you will receive**

You will initially meet an ACCESS Clinician, a trained person who will assess your needs and goals, offer information about available services, link you to appropriate services at ACCESS Open Minds (INSERT SITE NAME) or, if needed, refer you to other services located elsewhere.

Throughout your follow-up at ACCESS Open Minds (INSERT SITE NAME), our team will meet with you regularly to help you feel better, to support you and to work with you in achieving your goals. If we refer you to another service, the ACCESS Clinician and/or the ACCESS team will continue to connect with you and provide occasional follow-up, as necessary.

**The role of your family and carers**

We believe that support from families and carers can play a valuable role in young people’s lives. We therefore encourage your family and carers to be involved in your follow-up at ACCESS Open Minds (INSERT SITE NAME). However, we respect a separate and confidential relationship with you and will not involve your family and carers if you do not want us to. If you would like your family or carer to be involved in your follow-up, please share this letter with them.

**Why we conduct evaluations**

We will request you to participate in a few evaluations that can help us plan your care. These evaluations include questionnaires that you will be asked to fill out, either on a computer/tablet or on paper. These questionnaires can help us better assess and understand your needs and perspectives (e.g., how you see your health and well-being, your satisfaction with our services, how and where you looked for help, etc.).

These questionnaires will take about 10-15 minutes to complete and some of them will be repeated over the course of your follow-up to monitor your progress and understand changes in your needs and perspectives.

You will be able to discuss these evaluations with your ACCESS team at any time. All data collected about you and your family member/carers will remain confidential and available to you upon request.

**The ACCESS Open Minds research project**

ACCESS Open Minds is a national project being conducted at more than a dozen sites across Canada. Its objective is to improve the mental health of young people by making better care more easily available at the right time. As part of this project, we are evaluating whether the transformed ACCESS Open Minds services work as they are supposed to and how young people like you benefit from them. This evaluation will help decide if similar services should be made available everywhere across the country.

**How you can contribute**

If you consent, the evaluations you complete as part of your own care will be included in the ACCESS Open Minds research project. You are free to agree or to refuse to have your evaluations included in the ACCESS Open Minds research.

If you agree, you can change your mind at any time simply by telling us. Your decision will not impact the care that you receive or are entitled to receive.

As part of the evaluation of outcomes, we also require your permission to use your follow-up files at ACCESS Open Minds (INSERT SITE NAME). From these files, we will be able to record the time and steps it took for you to be referred, the time it took for you to receive an initial evaluation, the services you received, how you are doing, etc.

**Benefits**

You will not receive any direct benefits, money or other form of payment for consenting to ACCESS Open Minds’ research. However, by consenting, you will help us evaluate the impact of ACCESS Open Minds. The more people participate in this research, the better we will be able to understand the mental health care needs of young people and what works for them. Over the long term, this will help design mental health services that work better for young people like you.

**Risks**

Participating in ACCESS Open Minds research does not come with any risks or additional inconveniences.

**Confidentiality**

To protect your identity, results from your evaluations will be coded using unique numbers. Information that can identify you (e.g. name, address) will remain confidential and will never be made public. The data from your evaluations will be stored in a secure database on a protected server. All personal information will be destroyed *15* years after the end of the ACCESS Open Minds research project.

Findings from the evaluations of youth and families/carers who consent to participating in the ACCESS Open Minds research project may be included in scientific publications or discussions. In no such publication or discussion will it be possible to identify you or your family members/carers.

**Additional information**

*An independent research ethics board at the Douglas Mental Health University Institute in Montreal has approved the* ACCESS Open Minds research project. Any changes in this research require the ethics board’s further approval.

To know more about ACCESS Open Minds’ research, simply ask for our information document. Before you sign this form, take the time to read it and the information document (if you asked for one) and ask any questions you may have.

If you have any questions or concerns relating to your follow-up, your participation in evaluations or your participation in research, please contact: INSERT SITE LEAD and ACCESS co-applicant NAME, EMAIL AND TELEPHONE NUMBER. If you have a complaint, you may contact *[name and address].*

**Welcome**

We welcome you to our program and look forward to working with you towards the well-being that you desire. We invite you to share your suggestions on how we can best meet your needs and improve as a program, and to ask us any questions you may have throughout your follow-up in our program.

On behalf of ACCESS Open Minds (INSERT SITE NAME),

_________________________________
INSERT Name site lead and ACCESS co-applicant

**YOUTH PARTICIPANT CONSENT**
I have read and understood the above information and had the opportunity to ask questions. I understand that all data collected about me will remain confidential. Please place a check mark (✓) in the boxes next to the statement(s) that you consent to or agree with, as applicable.

**Receiving care**

- I agree to being offered services by ACCESS Open Minds (INSERT SITE NAME). I understand that I will be requested to complete evaluations that will help assess my well-being and plan my care
- I would like my family member/carer to be involved in the services I am offered at ACCESS Open Minds (INSERT SITE NAME).

**Participation in research on improving youth services**

I agree that data about my well-being and health, and services I have used can be included in the research that ACCESS Open Mind’s is conducting to improve mental health services for young Canadians. Specifically, I agree to data being collected from the following sources:

- Confidential responses to questionnaires/evaluations related to my care
- Clinically relevant current and previous data from charts and databases at services I have used, including at ACCESS Open Minds (INSERT SITE NAME)
- Administrative databases maintained by health and social services systems (e.g., INSERT NAME of provincial health care system like RAMQ or OHIP)

**Options regarding consent**

- If I stop using ACCESS Open Minds (INSERT SITE NAME)’s services, I agree that ACCESS Open Minds can use data from all sources that I have consented to.
- I agree that ACCESS Open Minds can use collected data to answer future questions related to improving youth mental health services in Canada

**Contact by email (through secure link)**

- I agree to being contacted by email only in relation to my participation in ACCESS Open Minds (INSERT SITE NAME)’s services and evaluations (e.g., to provide me with a secure link where I can access the self-report questionnaires online).

If yes, my email address is: ____________________________________________________ (please print)

A signed and dated copy of this consent form will be given to me.

__________________________________________

Printed name of youth

__________________________________________ _________________________________
Signature of youth Date

__________________________________________
Signature of the parent or guardian (if youth is under 14, or if youth is 14-17 and parent or guardian is involved in care)

**Declaration of the person obtaining consent** I confirm that the above information/consent form was explained to the youth; that questions that the youth had were answered; and that it was clearly indicated that the youth remains free to choose to not participate in evaluations and/or to not allow completed evaluations or their data to be included as part of ACCESS Open Minds' research, without any impact on services or care that the youth and his/her family/carers are entitled to receive.

I commit myself, as well as the ACCESS Open Minds (Insert site name) team I represent, to respect what was agreed upon in the information/consent form and to give a signed copy of this form to the youth.

**Printed name of person obtaining the consent:**

___________________________________________

____________________________ ____ ___________________________

*Signature of person obtaining consent* Date

**FAMILY MEMBER/CARER CONSENT (only for family members/carers above the age of 18)**

I have read and understood the above information and had the opportunity to ask questions. I understand that all data collected about me will remain confidential. Please place a check mark (✓) in the boxes next to the statement(s) that you consent to or agree with as applicable.

**Receiving Care**

- I agree to being offered services by ACCESS Open Minds (INSERT SITE NAME). I understand that I will be requested to complete evaluations that will help assess my perspectives.

**Participation in research on improving youth services**

- I agree that the evaluations I complete can be included in the research that ACCESS Open Mind’s is conducting to improve mental health services for young Canadians and their families/carers.

**Contact by email (through secure link)**

- I agree to being contacted by email only in relation to my participation in ACCESS Open Minds (INSERT SITE NAME)’s services and evaluations (e.g., to provide me with a secure link where I can access the self-report questionnaires online).

If yes, my email address is: ____________________________________________________ (please print)

A signed and dated copy of this consent form will be given to me.

__________________________________________

Printed name of family member/carer participant

__________________________________________ ________________________________
Signature of family member/carer participant Date

**Declaration of the person obtaining consent** I confirm that the above information/consent form was explained to the family member/carer; that questions that they had were answered; and that it was clearly indicated that the family member/carer remains free to choose to not participate in evaluations and/or to not allow completed evaluations to be included as part of ACCESS Open Minds' research, without any impact on services or care that they and his/her family member/loved one are entitled to receive.

I commit myself, as well as the ACCESS Open Minds (Insert site name) team I represent, to respect what was agreed upon in the information/consent form and to give a signed copy of this form to the youth and their family member/carer.

**Printed name of person obtaining the consent:**

___________________________________________

_______________________________ _______________________________

*Signature of person obtaining consent* Date

**ANNEX 1 - MODIFICATIONS TO YOUTH PARTICIPANT CONSENT**

I have read and understood the above information and had the opportunity to ask questions. I understand that all data collected about me will remain confidential. Please place a check mark (✓) in the boxes next to the statement(s) that you consent to.

**Modification to existing consent**

- I would like to change the terms of my previous consent dated ______________________. I understand that the statements I am making below will replace my previous selections.

**Participation in research on improving youth services**

I agree that data about my well-being and health, and services I have used can be included in the research that ACCESS Open Mind’s is conducting to improve mental health services for young Canadians. Specifically, I agree to data being collected from the following sources:

- Confidential responses to questionnaires/evaluations related to my care
- Clinically relevant current and previous data from charts and databases at services I have used, including at ACCESS Open Minds (INSERT SITE NAME)
- Administrative databases maintained by the health and social services systems (e.g., INSERT NAME of provincial health care system like RAMQ or OHIP)

**Options regarding consent**

- If I stop using ACCESS Open Minds (INSERT SITE NAME)’s services, I agree that ACCESS Open Minds can use data from all sources that I have consented to.

**Contact by email (through secure link)**

- I agree to being contacted by email only in relation to my participation in ACCESS Open Minds (INSERT SITE NAME)’s services and evaluations (e.g., to provide me with a secure link where I can access the self-report questionnaires online).

If yes, my email address is: ____________________________________________________ (please print)

A signed and dated copy of this consent form will be given to me.

__________________________________________

Printed name of youth

__________________________________________ _________________________________
Signature of youth Date

__________________________________________
Signature of the parent or guardian (if youth is under 14, or if youth is 14-17 and parent or guardian is involved in care)

**Declaration of the person obtaining consent** I confirm that the above information/consent form was explained to the youth; that questions that the youth had were answered; and that it was clearly indicated that the youth remains free to choose to not participate in evaluations and/or to not allow completed evaluations or their data to be included as part of ACCESS Open Minds' research, without any impact on services or care that the youth and his/her family/carers are entitled to receive.

I commit myself, as well as the ACCESS Open Minds (Insert site name) team I represent, to respect what was agreed upon in the information/consent form and to give a signed copy of this form to the youth.

**Printed name of person obtaining the consent:**

___________________________________________

____________________________ ____ ___________________________

*Signature of person obtaining consent* Date
