# Supplementary material for: A minimum evaluation protocol and stepped-wedge cluster randomized trial of ACCESS Open Minds, a large Canadian youth mental health services transformation project
Source: BMC Psychiatry. 2019 Sep 5;19:273. doi: 10.1186/s12888-019-2232-2 (PMC6729084; doi:10.1186/s12888-019-2232-2)
Supplement: Supplementary file 3 — Psychometric properties of study instruments. (DOCX 20 kb) [file 12888_2019_2232_MOESM3_ESM.docx]

Additional file 3: Psychometric properties of study instruments

| **Instrument** | **Reporter** | **Validity** | **Reliability** |
| --- | --- | --- | --- |
| Kessler-10 (K-10) | Youth | DSM-IV diagnosis: AUC = 0.85-0.96  Score of ≥30: sensitivity = 0.24 and specificity = 0.99 | α = 0.92-0.93 |
| Self-Rated Mental Health (SRMH) | Youth | *r* = 0.49 (K6)  *r* = 0.46 (SF-12)  *r* = 0.45 (PHQ-2) |  |
| Self-Rated Health (SRH) | Youth | *r* = 0.55-0.58 (SF-12) | kappa = 0.43 (between first and second administration) |
| Outcome Rating Scale (ORS) | Youth | Total: *r* = 0.53 – 0.74 (OQ-45)  Individual: *r* = 0.53-0.74 (OQ-45)  Distress: *r* = 0.53-0.74 (OQ-45)  Social: *r* = 0. 34-0.55 (OQ-45) | α = 0.90- 0.93  *r* = 0.54-0.80 (between baseline and second assessment) |
| Session Rating Scale  (SRS) |  | Total: *r* = 0.58 (WAI)  Relationship: *r* = 0.37 (WAI)  Goal and topics: *r* = 0.60 (WAI) | α = 0.88 – 0.93  *r* = 0.70 (between baseline and second assessment) |
| Youth Efficacy / Empowerment Scale (YES) | Youth | Higher scores among youth more satisfied with their treatment plans compared to less satisfied. | Self: α = 0.852  Services: α = 0.833  System: α = 0.882 |
| WHO Quality of Life (WHO-QOL-BREF) | Youth | WHO-QOL-BREF domains with their respective counterparts in the WHO-QOL  Comparative index = 0.90 (each comparison) | Physical α = 0.80-0.84  Psychological α = 0.75- 0.81  Environmental α = 0.80  Social α = 0.66-0.69 |
| Ontario Perception of Care Tool (OPOC) | Youth | Access: *r* = 0.49 (CSQ)  Services provided: *r* = 0.56 (CSQ) Participation/rights: *r* = 0.51 (CSQ) Therapists/support workers/staff: *r* = 0.54 (CSQ)  Environment: *r* = 0.50 (CSQ) Recovery/outcome: *r* = 0.54 (CSQ)  Service quality: *r* = 0.62 (CSQ)  Discharge: *r* = 0.49 (CSQ) | Access: α = 0.87  Services provided: α = 0.89  Participation/rights: α = 0.87  Therapists/support workers/staff: α = 0.92  Environment: α = 0.89  Discharge: α = 0.91  Overall experience: α = 0.91 |
| Social and Occupational Functioning Assessment Scale (SOFAS) | Clinician | *r* = 0.60-0.93 (GAF)  *r* = 0.34 (GARF)  *r* = 0.39-0.47 (SAS)  *r* = 0.85 (SAS social functioning subscale)  *r* = 0.58 (Strauss Carpenter Scale) | Inter-related reliability (Intraclass coefficient = 0.89-0.94) |
| Clinical Global Impressions (CGI) | Clinician | *r* = 0.71 (indirect improvement and CGI Improvement) | *r* = 0.40 (admission and discharge) |
| Columbia Suicide Severity Rating Scale (C-SSRS) | Clinician | Accurate in identify cases and non-cases demonstrating any suicidal behaviour within 6 months of discharge from a hospital (AUC = 0.76)  Total: *r* = 0.38-0.47 (BSSI)  Severity: *r* = 0.51-0.80 (BDI); *r* = 0.69 (SSI) | α = 0.73-0.95  kappa = 0.66 (Columbia Suicide History Form) |
| Chemical Use/Abuse/  Dependence (CUAD) | Clinician |  |  |
| Global Appraisal of Individual Needs – Short Screener (GAIN-SS) | Youth with Clinician | Total disorder screener: r = .94 with GAIN-I Severity  Internalizing: r = 0.89 with GAIN-I Internal  Externalizing: r = 0.88 with GAIN-I Behavior  Substance use: r = 0.92 with GAIN-I Substance  Crime/violence: r = 0.86 with GAIN-I Crime/Violence | Total disorder screener: α = 0.87  Internalizing: α = 0.74  Externalizing: α = 0.76  Substance use: α = 0.76  Crime/violence: α = 0.72 |

**References**

**K10**

Kessler RC, Andrews G, Colpe LJ, Hiripi E, Mroczek DK, Normand SL, et al. Short screening scales to monitor population prevalences and trends in non-specific psychological distress. *Psychol Med* 2002; **32**: 959-76.

Kessler RC, Barker PR, Colpe LJ, Epstein JF, Gfroerer JC, Hiripi E, et al. Screening for serious mental illness in the general population. *Arch Gen Psychiatry* 2003; **60**: 184-9.

Andrews G, Slade T. Interpreting scores on the Kessler Psychological Distress Scale (K10). *Aust NZ K Public Health* 2001; **25**: 494-7.

**SRMH**

Fleishman JA, Zuvekas SH. Global self-rated mental health: associations with other mental health measures and with role functioning. *Med Care* 2007; **45**: 602-9.

**SRH**

Zajacova A, Dowd JB. Reliability of self-rated health in US adults. *Am J Epidemiol* 2011; **174**: 977-83.

**ORS**

Miller SD, Duncan BL, Brown J, Sparks JA, Claud DA. The Outcome Rating Scale: A preliminary study of reliability, validity, and feasibility of a brief visual analog measure. *Journal of Brief Therapy* 2003; **2**: 91-100.

Bringhurst DL, Watson CW, Miler SD, Duncan BL. Outcome Rating Scale: A replication study of a brief clinical measure. *Journal of Brief Therapy* 2006; **5**: 23-30.

Duncan BL, Sparks JA, Miller SD, Bohanske RT, Claud DA. Giving youth a voice: A preliminary study of the reliability and validity of a brief outcome measure for children, adolescents and caretakers. *Journal of Brief Therapy* 2006; **5**: 71-88.

Campbell A, Hemsley S. Outcome Rating Scale and Session Rating Scale in psychological practice: Clinical utility of ultra-brief measures. *Clinical Psychologist* 2009; **13**: 1-9.

**SRS**

Hafkenscheid A, Duncan BL, Miller SD. The Outcome and Session Rating Scales: A cross-cultural examination of the psychometric properties of the dutch translation. *Journal of Brief Therapy* 2010; **7**: 1-12.

Campbell A, Hemsley S. Outcome Rating Scale and Session Rating Scale in psychological practice: Clinical utility of ultra-brief measures. *Clinical Psychologist* 2009; **13**: 1-9.

Duncan BL, Miller SD, Sparks JA, Claud DA, Reynolds LR, Brown J, et al. The Session Rating Scale: Preliminary psychometric properties of a “working” alliance measure. *Journal of Brief Therapy* 2004; **3**: 3-12.

**YES**

Walker JS, Powers LE. Introduction to the Youth Self-Efficacy Scale/Mental Health and the Youth Participation in Planning Scale. Portland, OR: Research and Training Center on Family Support and Children's Mental Health, Portland State University, 2007.

**WHO-QOL-BREF**

The WHOQOL Group. Development of the World Health Organization WHOQOL-BREF quality of life assessment. *Psychol Med* 1998; **28**: 551-8.

Skevington SM, Lofty M, O’Connell KA, WHOQOL Group. The World Health Organization’s WHOQOL-BREF quality of life assessment: psychometric properties and results of the international field trial. A report from the WHOOQOL group. *Qual Life Res* 2004; **13**: 299-310.

**OPOC**

Rush B, Hansson E, Cvetanova Y, Rotondi N, Furlong A, Behrooz R. Development of a client perception of care tool for mental health and addictions: Qualitative, quantitative, and psychometric analysis. Final Report for the Ministry of Health and Long‐Term Care. Toronto, Ontario: Health Systems and Health Equity Research, Centre for Addiction and Mental Health, 2013.

**SOFAS**

Hilsenroth MJ, Ackerman SJ, Blagys MD, Baumann BD, Baity MR, Smith SR, et al. Reliability and validity of DSM IV Axis V. *Am J Psychiatry* 2000; **157**: 1858-63.

Hay P, Katsikitis M, Begg J, De Costa J, Blumenfeld N. A two-year follow up study and prospective evaluation of the DSM-IV Axis V. *Psychiatr Serv* 2003; **54**: 1028-30.

Samara MT, Engel RR, Miller A, Kandenwein J, Toumi M, Leucht S. Equipercentile linking of scales measuring functioning and symptoms: examining the GAF, SOFAS, CGI-S, and PANSS. *Eur Neuropsychopharmacol* 2014; **24**: 1767-72.

Poirer S, Bureau V, Lehoux C, Bouchard RH, Maziade M, Pelletier S, et al. A factor analysis of the Strauss and Carpenter revised outcome criteria scale: a validation of the French translation. *J Nerv Ment Dis* 2004; **192**: 864-7.

**CGI**

Berk, M., Ng, F., Dodd, S., Callaly, T., Campbell, S., Bernardo, M., & Trauer, T. (2008). The validity of the CGI severity and improvement scales as measures of clinical effectiveness suitable for routine clinical use. *Journal of Evaluation in Clinical Practice*, *14*(6), 979-983.

**GAIN-SS**

Dennis ML, Chan YF, Funk RR. Development and validation of the GAIN Short Screener (GSS) for internalizing, externalizing and substance use disorders and crime/violence problems among adolescents and adults. Am J Addict. 2006;15 Suppl 1:80-91.

**C-SSRS**

Posner K, Brown GK, Stanley B, Brent DA, Yershova KV, Oquendo MA, et al. The Columbia-Suicide Severity Rating Scale: initial validity and internal consistency findings from three multisite studies with adolescents and adults. *Am J Pyschiatry* 2011; **168**: 1266-77.

Madan A, Frueh BC, Allen JG, Ellis TE, Rufino KA, Oldham JM, et al. Psychometric reevaluation of the Columbia-Suicide Severity Rating Scale: Findings from a prospective, inpatient cohort of severely mentally ill adults. *J Clin Psychiatry* 2016; **77**: e867-73.

Al-Halabi S, Saiz PA, Buron P, Garrido M, Benabarre A, Jimenez E, et al. Validation of a Spanish version of the Columbia-Suicide Severity Rating Scale (C-SSRS). *Rev Psiquiatr Salud Ment* 2016; **9**: 134-42.
