# Supplementary material for: A minimum evaluation protocol and stepped-wedge cluster randomized trial of ACCESS Open Minds, a large Canadian youth mental health services transformation project
Source: BMC Psychiatry. 2019 Sep 5;19:273. doi: 10.1186/s12888-019-2232-2 (PMC6729084; doi:10.1186/s12888-019-2232-2)
Supplement: Supplementary file 4 — Listing of ethics boards; age of research consent and need for parental consent for each site in the ACCESS Open Minds project. (DOCX 15 kb) [file 12888_2019_2232_MOESM4_ESM.docx]

Additional file 4: Listing of ethics boards; age of research consent and need for parental consent for each site in the ACCESS Open Minds project

| **Site** | **Institutional ethics board** | **Age of consent and assent (both consent and assent are obtained in writing)** |
| --- | --- | --- |
| Dorval-Lachine- Lasalle | Research Ethics Board (REB) of the Douglas Mental Health University Institute (DMHUI) | Assent if youth is under 14, or if youth is 14-17 and parent or guardian is involved in care |
| Parc-Extension | Research Ethics Board (REB) of the Douglas Mental Health University Institute (DMHUI) | Assent if youth is under 14, or if youth is 14-17 and parent or guardian is involved in care |
| RIPAJ-Montréal Homeless Youth Network | Comité d’éthique de la recherche du CHUM | Assent if youth is under 14, or if youth is 14-17 and parent or guardian is involved in care |
| Sturgeon Lake First Nation | Research Ethics Board (REB) of the Douglas Mental Health University Institute (DMHUI) (along with community approval) | Assent if youth is under 14, or if youth is 14-17 and parent or guardian is involved in care |
| Cree Nation of Mistissini | Research Ethics Board (REB) of the Douglas Mental Health University Institute (DMHUI) (along with community approval) | Assent if youth is under 16, or 16-17 with parent or guardian involved in care |
| Chatham-Kent | Chatham-Kent Health Alliance Ethics Review Board | Assent if youth is under 14, or if youth is 14-17 and parent or guardian is involved in care |
| Edmonton | University of Alberta Health Research Ethics Board (Panel B: Health Research) | Assent for 17 and younger. Consent for 18+ |
| University of Alberta | Research Ethics Board 1, University of Alberta | Assent for 17 and younger. Consent for 18+ |
| Elsipogtog First Nation | Comité d’éthique de la recherche avec les êtres humains de l’Université de Moncton (along with community approval) | Assent for 17 and younger. Consent for 18+ |
| Caraquet, Acadian Peninsula | Comité d’éthique de la recherche avec les êtres humains de l’Université de Moncton | Assent for 17 and younger. Consent for 18+ |
| PEER Saint John | Comité d’éthique de la recherche avec les êtres humains de l’Université de Moncton | Assent for 17 and younger. Consent for 18+ |
| Puvirnituq | Research Ethics Board (REB) of the Douglas Mental Health University Institute (DMHUI) (along with community approval) | Assent for youth under 16, or 16-17 with parent or guardian involved in care |
| Ulukhaktok | Aurora Research Institute | Assent for youth 15 and younger. Consent for 16+ |
| Eskasoni First Nation | Eskasoni Health Advisory Committee, Eskasoni Health Board, Eskasoni Band Council | Assent if youth is under 14, or if youth is 14-17 and parent or guardian is involved in care |

*Note*: Overarching ethics approval for the entire project was provided by the Research Ethics Board of the Douglas Mental Health University Institute, which is the parent or host institute for this research project (DMHUI)
